# Technological advancements for surgical planning and intraoperative navigation in elbow arthroplasty: a systematic review

**DOI:** 10.1016/j.jseint.2026.101776

**Published:** 2026-08-05

**Authors:** Mark-Jan Vles, Daan Trinh, Arno Macken, Anna van der Windt, Denise Eygendaal, Bertram The

**Affiliations:** a Department of Orthopaedic Surgery, Amphia Hospital, Breda, The Netherlands; b Department of Orthopaedics and Sports Medicine, Erasmus MC, University Medical Center Rotterdam, Rotterdam, The Netherlands

**Keywords:** Elbow arthroplasty, Surgical planning, Augmented reality, Robotics, Patient-specific instrumentation, Three-dimensional planning

## Abstract

**Background:**

Due to the complex load transmission across the elbow and the limited systems available for total elbow arthroplasty (TEA), optimal implant positioning, which is crucial for achieving satisfactory functional outcomes and longstanding implant survival, remains challenging. Technological advancements in orthopedic surgery are rapidly evolving, with some of the main goals including improving implant positioning. In contrast to more common arthroplasties, these techniques are rarely described for TEA. The purpose of this review is to assess the current technologies available for TEA and investigate whether these are beneficial for surgical accuracy, functional outcomes, and implant survival.

**Methods:**

A systematic search using Embase, Medline, Web of Science, Cochrane, and Google Scholar was used to identify articles investigating the use of novel technologies in surgical planning for TEA.

**Results:**

A total of 16 articles, published between 2007 and 2022, were included in this systematic review. Augmented reality, robotic tracking systems, patient-specific instrumentation, and 3-dimensional planning or printing were investigated in a total of 89 patients and 55 cadaver elbows. Compared to conventional methods, implant positioning was significantly improved using these novel technologies with similar short-term functional outcomes.

**Conclusion:**

Despite being sparsely investigated, several novel technologies are nowadays available for TEA. These technologies show improved implant positioning following TEA when compared to conventional planning methods. Although superior implant positioning did not directly translate into significantly better clinical outcomes, further investigation of the effect on long-term outcomes and implant survival is needed to assess the potential benefit of these techniques in future TEA.

Total elbow arthroplasty (TEA) is increasingly used as a treatment for end-stage joint degeneration due to rheumatoid arthritis or (post)traumatic causes, or in cases of comminuted fractures not amenable for fixation in the elderly.^47^ The complex forces within the elbow, limited articular exposure in most surgical approaches, challenging or absent anatomical landmarks, and scarcity of perioperatively instrument systems make optimal implant positioning for TEA challenging.^17^^,^^21^^,^^38^ Additionally, since elbow implants have limited versatility and sizing options compared to other arthroplasties, intraoperative modifications are relatively difficult. Malalignment or undersizing of either the ulnar, radial, or humeral component in TEA can lead to abnormal joint mechanics, potentially resulting in accelerated wear, post-operative instability, early loosening, and impaired implant survival and functional outcomes.^6^^,^^21^^,^^33^^,^^38^ Similar associations have been reported in more commonly performed joint arthroplasties, such as the hip, knee, and shoulder.^30^^,^^53^^,^^56^ Furthermore, adequate cement-mantle quality and cement positioning are associated with lower rates of humeral component loosening.^26^ The inferior 10-year implant survival following TEA compared to more frequently performed arthroplasties such as the hip or knee underscores the necessity of proper implant and cement positioning.^4^^,^^14^^,^^49^^,^^55^^,^^58^

In terms of pre-operative planning, orthopedic surgeons have traditionally relied on conventional 2-dimensional (2D) radiographs. It has been proven that this technique is insufficient in obtaining an accurate pre-operative surgical plan.^48^ Nowadays, technological advancements in orthopedic surgery are rapidly evolving, with improved implant positioning as one of the main goals.^10^ These novel techniques include extended reality (ER), robotics, patient-specific instrumentation (PSI), 3-dimensional (3D) pre-operative planning methods, and customized printed implants (CPI).^3^^,^^10^

ER is a technology in which the physical and digital worlds are blended.^10^ This includes several techniques such as virtual reality (VR) and augmented reality (AR). VR, first introduced in the 1950s, is an immersive 3D-generated experience with realistic images and sounds.^59^ AR, introduced in 1997, differs from VR due to the incorporation of the real-world environment.^1^^,^^59^ This is usually achieved by overlaying digital information onto objects in the real world using a head-mounted display with see-through glasses.^2^ The potential use of AR in medicine was first mentioned in 1997 and has since been extensively studied across multiple surgical disciplines.^50^^,^^51^^,^^63^

Robotic-assisted technology for orthopedic surgery was first used in 1992 for total hip arthroplasty.^42^ It consists of a robotic system that is used during surgery for enhanced surgical navigation and implant positioning.^3^ Current evidence indicates that the most important disadvantages of robotic-assisted surgery are the high costs of such a system and the increased surgical operation time, while post-operative complication risks and patient-reported outcome measures are similar when compared to conventional methods.^3^^,^^12^

For PSI, pre-operative magnetic resonance imaging (MRI) or computed tomography (CT) scans are used to create a 3D model in which the implant position is planned.^66^ Based on this pre-operative plan and patient anatomy, an individualized osteotomy guide is printed. PSI was first introduced in the early 2000s as a guide for drilling pedicle screws but is nowadays increasingly used during surgery to achieve a proper implant positioning.^15^^,^^16^^,^^27^^,^^52^^,^^66^

CPIs are patient-specific, 3D-printed titanium implants designed using a pre-operative CT-based 3D model. They are most frequently used in oncologic reconstructions when standard implants do not adequately match patients' anatomy.^13^^,^^34^ However, they have also been used for complex elbow fractures, in which a mirrored CT-based model of the contralateral elbow can be used to fabricate a customized implant.^13^

Lastly, 3D pre-operative planning enables the creation of highly accurate 3D reconstructions of patient-specific elbow anatomy.^28^^,^^39^ These digital models, based on CT or MRI scans, enable virtual pre-operative planning and simulation of implant placement, potentially improving surgical accuracy and reducing the risk of complications.^28^^,^^39^

All these novel techniques have been extensively investigated for other types of arthroplasties and show variable results in terms of functional outcomes.^5^^,^^15^^,^^16^^,^^18^^,^^27^^,^^31^^,^^32^^,^^44^^,^^64^ However, for TEA, these technologies have rarely been described but tend to show an association with improved implant positioning.^28^^,^^39^^,^^40^^,^^66^ The integration of such image-based planning methods into clinical workflows could potentially enhance functional outcomes and implant survival following TEA.

This systematic review aims to investigate whether these innovative technologies are beneficial for TEA in terms of surgical accuracy, complications, functional outcomes, and implant survival.

## Methods

This systematic review was conducted in accordance with the Preferred Reporting Items for Systematic Reviews and Meta-Analyses guidelines.^54^

### Search strategy

On November 6, 2025, a systematic search string was conducted by a specialized search coordinator using Embase, Medline (Ovid), Web of Science, Cochrane, and Google Scholar. Articles had to be written in either French, German, Dutch, or English and were published between 2007 and 2025. A detailed search strategy for all databases is provided in Supplementary Table 1. Reference tracking (snowballing) was used to identify any additional studies.^23^ Human or cadaver studies elaborating on surgical planning, total or hemi elbow arthroplasty, and the use of novel technologies such as VR, AR, PSI, robotics, or 3D planning were included. Articles were excluded if they did not describe any intraoperative or post-operative outcome.

All articles were checked for duplicates. Using predefined criteria and definitions, eligibility was assessed by 2 independent authors (M.V. and D.T.) by scanning the title and abstract and the full text if necessary. Disagreement was resolved with discussion. If disagreement persisted, a third author (A.M.) was consulted. Covidence systematic review software was used for the selection process (Veritas Health Innovation, Melbourne, Australia; available at https://www.covidence.org/).

### Outcomes

The primary outcomes were post-operative elbow function, patient-reported outcomes, and implant survival following TEA. Secondary outcomes were accuracy of implant position, complications, duration of surgery, and costs.

### Definitions

VR was defined as a 3D-generated virtual world in which the user was able to look around with the use of a head-mounted display. AR was specified as a technique in which digital information was projected over objects in the real world. PSI was defined as any physical type of patient-specific intraoperative guide or instrumentation. If a robotic arm was used during the surgery in any way, it was categorized as robotics. CPIs were defined as a patient-specific 3D-printed implant used for partial hemiarthroplasty, total hemiarthroplasty, or TEA. Lastly, 3D planning was categorized as a digital or physical 3D model of patient-specific elbow anatomy, which was used for planning implant position. All these techniques had to be used for surgical planning in either the pre-operative or intraoperative phase.

### Quality assessment

After inclusion of the articles, study design was identified, and the corresponding quality assessment was carried out by 2 independent authors (M.V. and D.T.). Randomized controlled trials were scored using the Delphi Verhagen Checklist.^62^ Cohort or case-control studies were graded using the Newcastle-Ottawa Scale.^57^ Any case series or case reports were assessed using the checklist described by Murad et al.^43^ As proposed by Murad et al^43^, an overall judgment about methodological quality within each domain was made instead of an aggregate numeric score. The article was considered of high methodological quality if the overall judgment was positive for all 4 domains. If the overall judgment was negative for at least 2 domains, the study was considered of poor methodological quality. All remaining articles were considered of moderate methodological quality. Any disagreement was resolved through discussion, and if disagreement persisted via consulting the senior author (B.T.).

### Statistical analysis

Descriptive statistics were used to summarize data. Continuous variables are presented using the mean and corresponding standard deviation (SD). If the SD was unavailable or could not be calculated, we reported the mean with the corresponding range. Categorical variables are presented as absolute counts with corresponding percentages.

## Results

**Figure 1:**
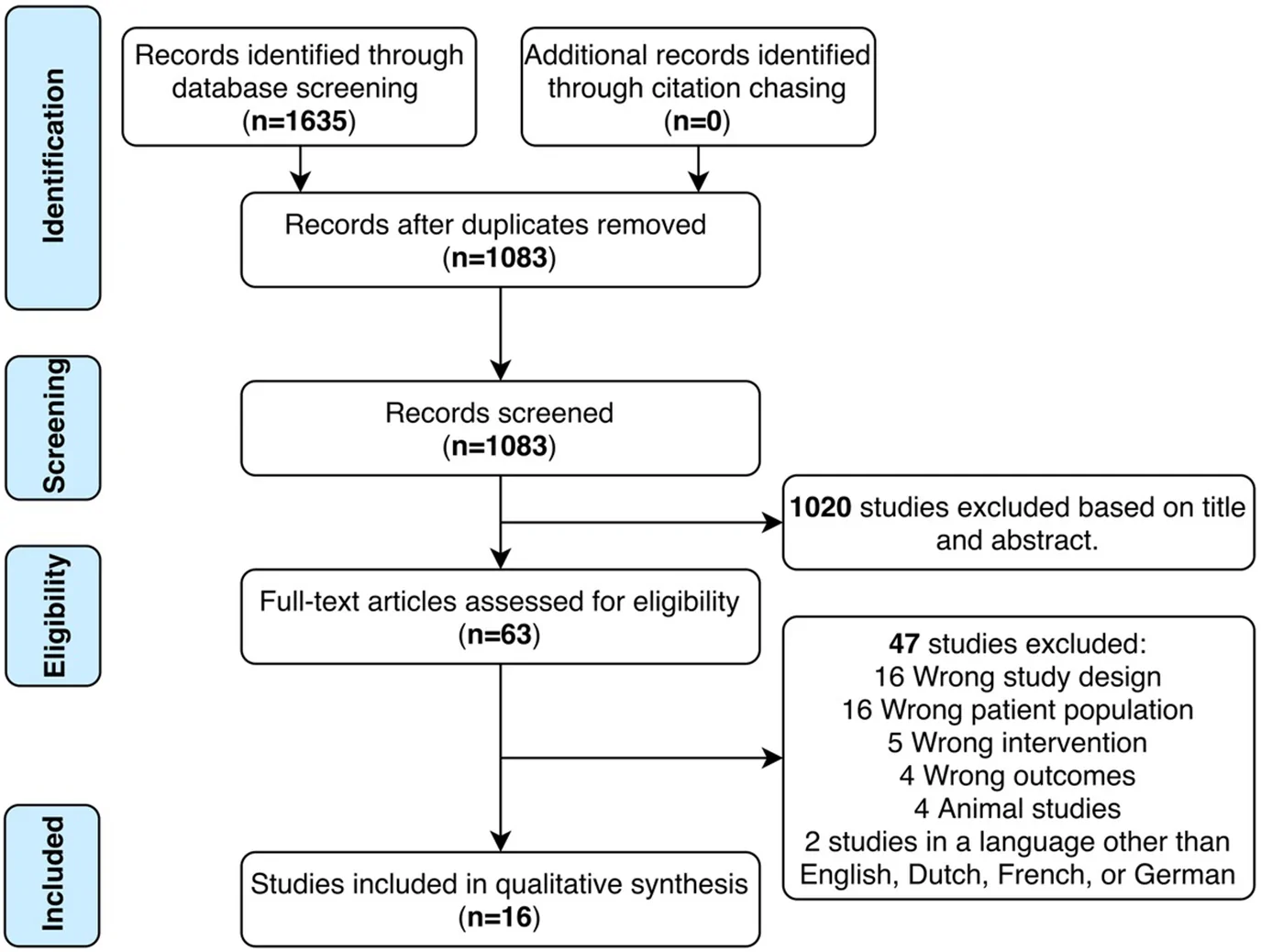
Flow diagram of study selection process. Preferred Reporting Items for Systematic Reviews and Meta-Analyses (PRISMA) flow diagram.

The literature search yielded a total of 1635 articles. After duplicate removal and screening, a total of 63 articles were assessed for full-text review. Eventually, 16 articles were included in this systematic review (Fig. 1). No additional articles were identified through citation chasing.

### Study characteristics

There were 3 retrospective case series and 2 retrospective case-control studies. The remaining studies were either cadaver studies or case reports. A total of 89 patients, 55 cadaver elbows, and one patient-based 3D-printed elbow were studied (Table I).^7^^,^^9^^,^^13^^,^^25^^,^^28^^,^^34^^,^^35^^,^^39^, ^40^, ^41^^,^^46^^,^^60^^,^^65^, ^66^, ^67^, ^68^

**Table I:** Overview of included studies.

Author year	Study design	Implant model	Procedure	Population	Intervention	Outcomes
Brownhill et al 2007^9^	*In vitro* study	U: Latitude TEA H: Custom	TEA	Cadaver elbows N = 8	EMT system	Implant positioning
McDonald et al 2010^40^	*In vitro* study	Latitude TEA	TEA	Cadaver elbows N = 11	EMT system	Implant positioning
McDonald et al 2011^41^	*In vitro* study	Latitude TEA	TEA	Cadaver elbows N = 13	EMT system	Implant positioning
Brownhill et al 2012^7^	*In vitro* study	U: Latitude TEA H: Custom	TEA	Cadaver elbows N = 11	EMT system	Implant positioning
Iwamoto et al 2018^28^	Case series	Unlinked K-NOW	TEA	Human elbows N = 28	3D planning	Implant positioning Implant size estimation
Wu et al 2020^65^	Case report	Custom	TEA	Human elbow N = 1	3DP implant	ROM, MEPS
Heidari et al 2021^25^	*In vitro* study	Coonrad-Morrey	TEA	3D printed bone N = 1	PSI	Position error for the humeral canal
Liang et al 2022^34^	Case series	Custom	EHA	Human elbows N = 13	3DP implant	ROM, MEPS
Tanji et al 2022^60^	Case control	Unlinked K-NOW	TEA	Cadaver elbows N = 12	AR = 6 Conv = 6	Implant positioning
Zhu et al 2022^68^	Case report	Custom	TEA	Human elbow N = 1	3DP implant	ROM, MEPS
Liao et al 2023^35^	Case report	Custom	EHA	Human elbow N = 1	3DP implant and PSI	ROM, MEPS
Zhu et al 2023^67^	Case report	Custom	TEA	Human elbow N = 1	3DP implant	ROM, MEPS
Choo et al 2024^13^	Case report	Custom	EHA	Human elbow N = 1	3DP implant and PSI	Patient satisfaction
Matsuo et al 2024^39^	Case series	Unlinked K-NOW	TEA	Human elbows N = 22	3D planning	Implant positioning, ROM, VAS, MEPS
Pan et al 2025^46^	Case report	Custom	EHA	Human elbow N = 1	3DP implant and PSI	ROM, VAS, MEPS
Zhang et al 2025^66^	Case control	Unknown	TEA	Human elbows N = 20	PSI = 9 Conv = 11	Elbow axis, MEPS, Intraoperative fluoroscopy

*TEA*, total elbow arthroplasty; *EHA*, elbow hemiarthroplasty; *U*, ulnar; *H*, humeral, *EMT*, electromagnetic tracking, *3D*, three-dimensional; *3DP*, three-dimensional printed; *PSI*, patient-specific instrumentation; *AR*, augmented reality; *CONV*, conventional methods; *ROM*, range of motion; *VAS*, visual analog score for pain; *MEPS*, Mayo Elbow Performance Score.

### Three-dimensional pre-operative surgical planning

The potential use of 3D pre-operative planning was investigated by 2 retrospective case series from the same research group.^28^^,^^39^ The K-NOW (Teijn-Nakashima Medical, Okayama, Japan), which has an unlinked design, was used in a total of 50 TEA within both studies.^28^^,^^39^ Mean rotational and translational implant malposition was, apart from the rotational ulnar position error, within 4° and 2.2 mm, respectively.^28^^,^^39^ The mean rotational ulnar position error was 10 (SD, 7) but was not associated with impaired clinical outcomes.^39^ Two (10%) cases of aseptic loosening were reported.^39^ At 1-year follow-up, the mean Mayo Elbow Performance Score was 93.^28^^,^^39^ Elbow range of motion was more than 100° (Table II).^39^

**Table II:** Total elbow arthroplasty using pre-operative three-dimensional planning; outcomes.

Author	Sample size	Mean position error compared to pre-operative design	Estimated implant size compared to 2D planning	Mean ROM	Mean MEPS	Radiographic outcomes
Heidari et al 2021^25^	TEA (n = 28)	Ulnar: VV 1° (−4 to 4), FE: 2° (−1 to 10) Humeral: VV: 1° (−2 to 4), FE 2° (−1 to 4)	Ulnar: 96% (3D) vs. 68% (2D), *P* = .005 Humeral: 68% (3D) vs. 57% (2D), *P* = .018	E: −22° (−25 to 8)	92 (80 to 100)	Post-operative instability (n = 2), penetration of ulnar stem (n = 1), medial humeral condylar fracture (n = 1)
Matsuo et al 2024^39^	TEA (n = 22)	Ulnar: AP 1.5 mm (±1.7), RU 1.6 mm (±1.2), PD 1.6 mm (±1.7), FE -1° (±1), VV 1° (±1), EI 3° (±3) Humeral: AP 1.1 mm (±1.2), RU 1.5 mm (±1.4), PD 2.2 mm (±1.6), FE -4° (±2), VV 3° (±3), EI 10° (±7)	NA	F: 132° (±11) E: −19° (±17) P: 65° (±21) S: 66° (±25)	94 (±8)	Aseptic loosening of both components (n = 1), aseptic loosening of ulnar component (n = 1)

*ROM*, range of motion; *MEPS*, Mayo Elbow Performance Score; *TEA*, total elbow arthroplasty; *2D*, two-dimensional, *AP*, anterior-posterior; *RU*, radial-ulnar; *PD*, proximal-distal; *FE*, flexion-extension; *VV*, varus-valgus; *EI*, external-internal rotation; *NA*, not available; *F*, flexion; *E*, extension; *P*, pronation; *S*, supination.

The study by Iwamoto et al found that implant size estimation was significantly improved with the use of 3D planning methods when compared to 2D planning, based on conventional anteroposterior and axial radiographs.^28^

### Augmented reality

The use of AR was evaluated in 1 cadaver study only.^60^ In this study, TEA, using an unlinked K-NOW implant, was performed on 6 elbows using AR and on 6 elbows using traditional methods and conventional imaging.^60^ Patient-specific markers were printed and fixed to the cortical bone of the humerus and ulna using Kirschner wires.^60^ By scanning these markers with a tablet, the pre-operatively planned implant position and osteotomy lines were superimposed onto the bone.^60^ The mean translational and rotational position error for both humeral (*P* = .*002* for both translation and rotation) and ulnar implants (*P* = .*004* for translation and *P* = .*002* for rotation) was significantly lower when AR was used compared to traditional methods (Table III).^60^

**Table III:** Total elbow arthroplasty using augmented reality and patient-specific instrumentation; outcomes.

Author	Sample size	Mean position error compared to pre-operative design	Intraoperative fluoroscopy times	Mean MEPS
Heidari et al 2021^25^	N = 1 PSI = 1 Control = NA	Hum base: AP -0.4 mm, RU -0.4 mm Hum apex: AP -0.5 mm, RU -0.4 mm	NA	NA
Tanji et al 2022^60^	N = 12 AR = 6 Control a = 6	Hum rotation: 1° ± 1 (AR) vs. 4° ± 0 (control), *P* = .002 Hum translation: 1.5 mm ± 0.6 (AR) vs. 8.6 mm ± 1.3 (control), *P* = .002 Uln rotation: 6° ± 3 (AR) vs. 20° ± 10 (control), *P* = .004 Uln translation: 1.5 mm ± 0.4 (AR) vs. 6.9 mm ± 1.6 (control), *P* = .002	NA	NA
Zhang et al 2025^66^	N = 22 PSI = 9 Control∗ = 11	NA	2.5 ± 0.2 (PSI) vs. 5.8 ± 0.2 (control), *P* < .001	90.6 ± 0.5 (PSI) vs. 88.3 ± 0.5 (control), *P* > .05

*MEPS*, Mayo Elbow Performance Score; *PSI*, patient-specific instrumentation; *AR*, augmented reality; *Hum*, humeral; *Uln*, ulnar; *AP*, anterior-posterior; *RU*, radial-ulnar; *NA*, not available.

∗ Traditional techniques and conventional planning methods are used for the control group.

### Patient-specific instrumentation

A retrospective case-control study from Zhang et al compared the outcomes of patient-specific osteotomy guides to traditional methods for TEA.^67^ Intraoperative fluoroscopy times were significantly less using PSI compared to traditional methods (*P* < .*001*). However, patient-reported outcomes were similar (Table III).^67^ Also, there was 1 *in vitro* study in which a PSI was fabricated for drilling the humeral canal of a Coonrad Morrey implant (Zimmer Biomet, Warsaw, Indiana).^25^ This patient-specific drill guide was tested on a 3D printed humerus.^25^ The mean position error of the drilled humeral canal compared to the planned position was less than 0.5 mm.^25^

### Robotic electromagnetic tracking system

Four cadaver studies, using a total of 43 elbows, investigated the potential use of an electromagnetic tracking system for optimal implant positioning (Table IV).^7^^,^^9^^,^^40^^,^^41^ Two studies by McDonald et al assessed humeral implant positioning using the humeral component from the Latitude TEA (Tornier, Bloomington, Minneapolis).^40^^,^^41^ The 2 remaining studies assessed both humeral and ulnar implant positioning, using a customized humeral implant and an ulnar component from the Latitude TEA.^7^^,^^9^ Mean humeral translational and angular implant alignment was within 2 millimeters and 4°, respectively.^7^^,^^9^^,^^40^^,^^41^ For the ulnar component, translational position error was less than 2.3 mm for all directions, while the mean combined angular position error was 14° (SD7).^7^^,^^9^ Since all studies were cadaver studies, no clinical or patient-reported outcomes have been published to date.^7^^,^^9^^,^^40^^,^^41^

**Table IV:** Total elbow arthroplasty using an electromagnetic tracking system; outcomes.

Author	Sample size	Mean position error compared to pre-operative design
Brownhill et al. 2007^9^	N = 8	Hum: total rotation <1°, total translation <1 mm† Uln: RU 3.5 mm, PD -0.8 mm, VV 1° ± 4, EI 1° ± 9.0
McDonald et al 2010^40^	N = 11∗	Hum rotation (stage 1): 1° ± 0 (EMTS) vs. 5° ± 4 (control) Hum translation (stage 1): 1.2 mm ± 0.3 (EMTS) vs. 3.1 mm ± 1.3 (control) Hum rotation (stage 2): 2° ± 1 (EMTS) vs. 12° ± 3 (control) Hum translation (stage 2): 1.1 mm ± 0.5 (EMTS) vs. 3.0 mm ± 1.6 (control)
McDonald et al. 2011^41^	N = 13	Hum: total rotation <4° ± 2, total translation 1.9 mm ± 1.1†
Brownhill et al. 2012^7^	N = 11	Hum: AP 1.5 mm ± 1.2, RU -1.2 mm ± 1.4, PD 1.7 mm ± 2.1, VV 4° ± 1, EI 1° ±4 Uln: AP -0.9 mm ± 2.3, RU 1.5 mm ± 2.4, PD -0.8 ± 3.0, total rotation 14° ±7†

*Hum*, humeral; *Uln*, ulnar; *AP*, anterior-posterior; *RU*, radial-ulnar; *PD*, proximal-distal; *FE*, flexion-extension; *VV*, varus-valgus; *EI*, external-internal rotation; *EMT*, electromagnetic tracking.

∗ Non-navigated and navigated implant positioning was performed on the same cadaver elbow. Additionally, an intact distal humerus (stage 1) was compared to a distal humerus without anatomical landmarks (stage 2).

† Only the combined rotational or translational error was reported.

### Three-dimensional printing

Seven studies, encompassing a total of 19 patients, described the use of a custom printed implant based on a pre-operative CT-based 3D model (Table V).^13^^,^^34^^,^^35^^,^^46^^,^^65^^,^^67^^,^^68^ Indications for surgery were either a malignant lesion (n = 15) or a fracture of the distal humerus (n = 4).^13^^,^^34^^,^^35^^,^^46^^,^^65^^,^^67^^,^^68^ Two patients received a TEA,^65^^,^^67^ while the remaining patients received a (partial) humeral or ulnar hemiarthroplasty.^13^^,^^34^^,^^35^^,^^46^^,^^65^^,^^68^ In 3 studies, PSI was used to achieve a proper implant positioning.^13^^,^^35^^,^^46^ None of these studies assessed implant accuracy.^13^^,^^34^^,^^35^^,^^46^^,^^65^^,^^67^^,^^68^ Follow-up ranged from 6 months to 3 years.^13^^,^^34^^,^^35^^,^^46^^,^^65^^,^^67^^,^^68^ The study from Liang et al reported 4 complications, including 2 superficial wound problems, 1 temporary radial nerve palsy, and 1 dislocation.^34^ The remaining studies, which were all case reports, observed no complications.^13^^,^^35^^,^^46^^,^^65^^,^^67^^,^^68^ All patients were pain-free at last follow-up.^13^^,^^34^^,^^35^^,^^46^^,^^65^^,^^67^^,^^68^ Mean (range) flexion-extension arch and Mayo Elbow Performance Score were known for 18 patients and were 110 (60-135) degrees and 97 (85-100), respectively.^34^^,^^35^^,^^46^^,^^65^^,^^67^^,^^68^

**Table V:** Elbow arthroplasty using a three-dimensional printed implant; outcomes.

Author	Year	Sample size	Mean follow-up (months)	Mean ROM	Mean MEPS
Wu et al^65^	2020	TEA (N = 1)	36	FE 135°, PS 90°	90
Liang et al^34^	2022	EHA (N = 13) Humeral (N = 8) Ulnar (N = 5)	25.7 ± 7.8	FE: 108° ± 21, PS NA	97.7 ± 4.4
Zhu et al^67^	2022	TEA (N = 1)	15	FE 93°, PS 115°	86
Liao et al^35^	2023	EHA (N = 1)∗	6	FE 135°, PS NA	95
Zhu et al^68^	2023	TEA (N = 1)	12	FE 130°, PS NA	100
Choo et al^13^	2024	EHA (N = 1)∗	12	NA	NA†
Pan et al^46^	2025	EHA (N = 1)∗	6	FE 90°, PS NA	87

*ROM*, range of motion; *MEPS*, Mayo Elbow Performance Score; *TEA*, total elbow arthroplasty; *EHA*, elbow hemiarthroplasty; *FE*, flexion-extension; *PS*, pronation-supination; *NA*, not available.

∗ Partial humeral implant.

† MEPS was not reported, but patient was pain free at end of follow-up.

### Virtual reality

No articles on VR were found in the context of TEA.

### Quality assessment

**Figure 2:**
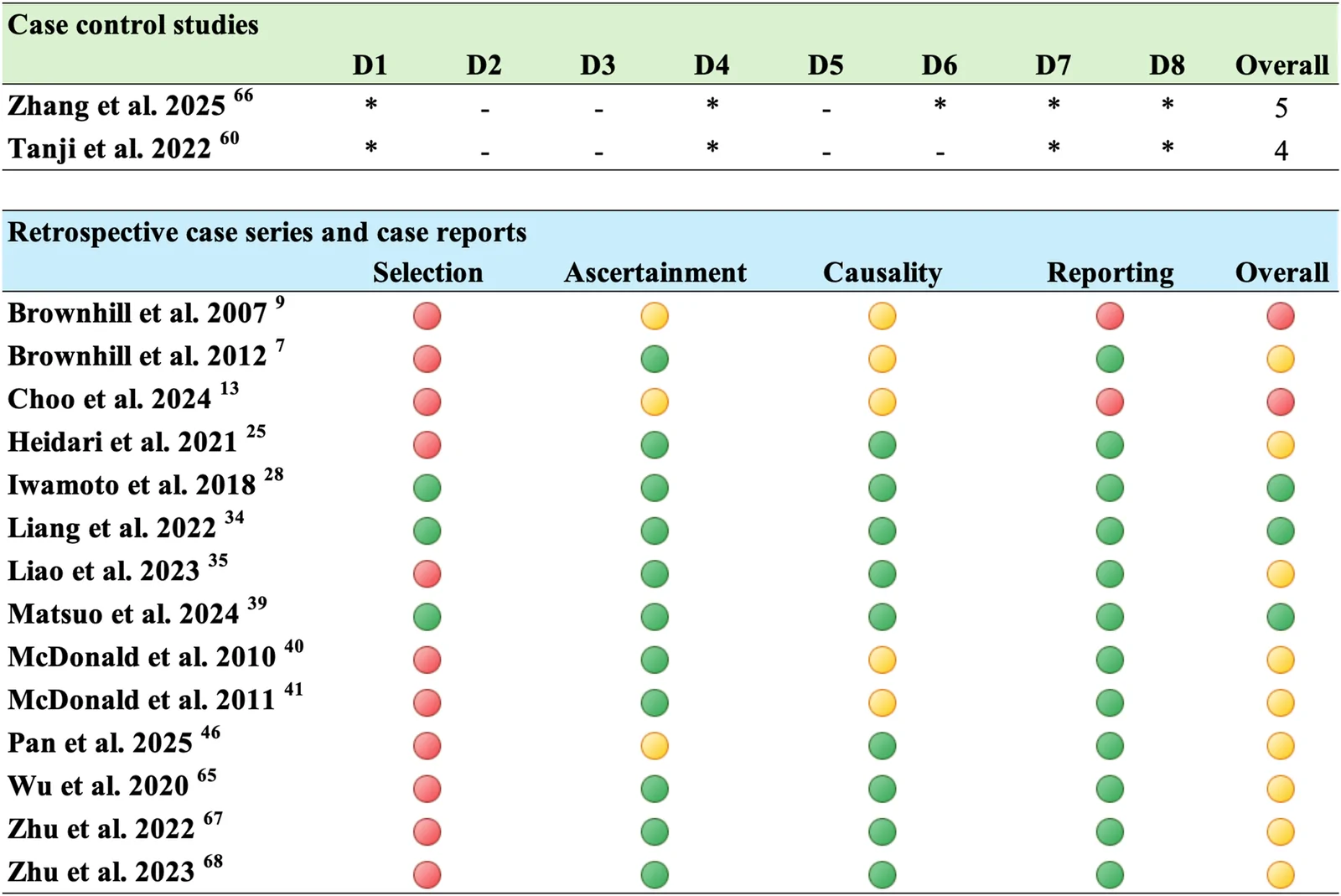
Risk of bias in individual studies. The Newcastle-Ottawa Scale was used for case-control studies, and the checklist described by Murad et al was used for retrospective case series and case reports. Study quality for case series and case reports is divided into good (*green*), moderate (*orange*), or poor (*red*) methodological quality.

Both case-control studies were graded as having a high rate of bias. The retrospective case series and case reports were most frequently graded as having a moderate methodological quality. The risk of bias assessment is reported in Figure 2.

## Discussion

In this systematic review, we elaborated on the potential use of several novel technologies, such as 3D planning, AR, and PSI, for surgical planning in TEA. Despite being sparsely investigated, and mostly in case reports or cadaver studies, these technologies show excellent implant positioning and satisfactory clinical and patient-reported outcomes.^7^^,^^9^^,^^25^^,^^28^^,^^39^, ^40^, ^41^^,^^53^^,^^59^

Among these technologies, only AR and an electromagnetic tracking system were associated with improved implant positioning compared to conventional methods.^40^^,^^60^ As these studies were cadaveric, no clinical outcome data are available for these techniques. However, in the few clinical studies in which either 3D planning or PSI was assessed, no association was found between implant positioning and patient-reported or clinical outcomes.^60^^,^^66^ This raises the question of the extent to which implant malalignment is associated with impaired functional outcomes. For TEA, achieving proper ulnar implant positioning remains most challenging, with mean rotational position errors of 10 and 6° for 3D pre-operative planning and AR, respectively.^39^^,^^60^ Matsuo et al found that malposition of the ulnar component, defined as a rotation error of more than 10°, was not associated with impaired short-term clinical outcomes when compared to a rotation error of less than 10°.^39^ Although short-term clinical outcomes are similar in these studies, prior evidence has indicated that ulnar component malpositioning with a valgus or varus alignment of more than 5° is associated with significantly worse long-term implant survival.^6^ It must be taken into account that these results are based on small case studies with a high risk of bias. Larger cohorts are required to assess the correlation between positioning, clinical outcomes, and implant survival. Also, since implant malalignment has been associated with increased component load, it could be hypothesized that longer follow-up times are required to develop increased component wear and potential impaired clinical outcomes after implant malalignment.^8^ The mean follow-up period for patients undergoing TEA using 3D pre-operative planning in this review was 47 months, which might have been an insufficient time for malalignment-induced component wear, resulting in impaired clinical outcomes or implant loosening.

Apart from implant positioning, the use of PSI was also associated with a lower amount of intraoperative fluoroscopy times, leading to a decrease in radiation exposure and potentially enhancing surgical efficiency.^66^

The study by Iwamoto et al found that implant size estimation for the unlinked K-NOW implant was significantly improved using 3D pre-operative planning software compared to conventional 2D radiographs.^34^ The clinical relevance of this finding remains questionable as the K-NOW Implant is available in only 3 different sizes, and the final implant size is selected by the orthopedic surgeon intraoperatively. Nevertheless, it still illustrates the increased accuracy of 3D planning compared to conventional planning methods.

Several studies described the use of a custom-printed implant, which was based on a pre-operative CT-based 3D model.^13^^,^^34^^,^^35^^,^^46^^,^^65^^,^^67^^,^^68^ Within these studies, excellent clinical and patient-reported outcomes were reported.^13^^,^^34^^,^^35^^,^^46^^,^^65^^,^^67^^,^^68^ This technique was most frequently used for malignant lesions or complex fractures. Still, it might also be useful for patients with other types of complex deformities or specific cases of revision surgery with extensive bone loss.

It is important to distinguish these findings from those in TEA, since elbow hemiarthroplasty was only investigated in the context of 3D-printed custom implants and exclusively in case series or case reports.^13^^,^^34^^,^^35^^,^^46^ In contrast, the use of AR, robotics, PSI, and 3D planning was solely studied in TEA. Given the difference in surgical indication, biomechanics, and failure modes between hemiarthroplasty and TEA, the current evidence does not allow direct translation of findings from TEA to elbow hemiarthroplasty and vice versa.^19^^,^^29^

Remarkably, no studies on the use of VR for pre- or intra-operative planning in TEA were identified. A possible explanation is that VR provides an immersive experience in which the user is visually and often auditorily isolated from the surrounding environment.^59^ This makes VR particularly suitable for educational purposes and surgical training, in which a surgical scenario can be created in a controlled setting.^36^ However, it is less practical for pre-operative planning and subsequent intraoperative navigation, where continuous awareness of the surgical field is essential.

Despite the potential benefits of these technologies, each of these novel innovations has its specific limitations that must be considered before adoption in clinical practice.

PSI, for instance, requires a pre-operative CT or MRI scan, after which the patient-specific guides are printed and sterilized. These guides are frequently produced outside the hospital, making them costly, time-consuming, and unsuitable for urgent cases. Proper intra-operative fixation of these guides may also require additional soft-tissue release during surgery, potentially increasing surgical duration and intra-operative complications. Lastly, the printed guides may become useless when any intraoperative events change the surgical plan.

Robotic surgery is, similar to PSI, costly and has been associated with increased operative time and a shallow learning curve.^45^ Robotic navigation also requires intraoperative guidance, usually achieved by inserting a navigation tracker into the bone. The holes drilled for these trackers can lead to a fracture or infection. A systematic review, investigating 33,724 robotic-assisted total knee arthroplasties, found a fracture rate at the pin site of 0.28% and a pin track infection rate of 2.7%.^45^ A similar risk can be imagined for robotic-assisted surgery in TEA.

ER aims to tackle the high costs of robotics and time-consuming production of PSI by giving the surgeon a wearable head-mounted display. VR is more suitable for pre-operative planning as it immerses the orthopedic surgeon in a virtual 3D-generated world, while AR is more promising for intraoperative use and is rather easily integrated into the normal surgical procedure.^11^ Key limitations are visual fatigue and unanticipated anatomical variations.^24^^,^^37^ One of the most important challenges for AR remains a precise calibration between the real environment and the visual overlay. Some studies, for instance, report slight deviations of the projected images when the headset is moved, resulting in an inaccurate visual overlay.^22^^,^^24^

One of the methods that does not require any additional intra-operative adjustment is 3D planning. However, it does require a pre-operative CT or MRI scan, after which a digital 3D model is made in which the implant position is planned. These 3D models are usually produced by technicians outside the hospital, after which the optimal implant position is coordinated with the performing surgeon, making it more costly and time-consuming. The accuracy of these models depends on the quality of the CT scan and does not take soft tissue into account. Using MRI scans or novel photon-counting CT scans for 3D model making could overcome this problem.^61^

Another general ethical consideration is that for the use of these technologies, patient data sometimes needs to be processed outside of the hospital. Sharing, storage, and processing of this data outside the hospital raises the question of patient privacy. Adherence to a secure data protection policy, such as the General Data Protection Regulation, is therefore paramount.^20^

This systematic review also has some limitations on its own, such as the lack of a meta-analysis due to the heterogeneity of the data in this study. Second, the small number of clinical studies and predominance of cadaveric or experimental studies limit conclusions regarding clinical endpoints. This limited sample reflects the fact that there are currently no widely available options for AR, PSI, and 3D planning in TEA. This highlights the novelty but also the limited focus and innovation of these techniques when applied to the elbow. Technological advancement is required to overcome several limitations, improve user-friendliness, and decrease costs. Further research, focusing on clinical outcomes, is warranted to assess the potential clinical benefits of these novel technologies in TEA.

## Conclusion

There are several novel technologies such as AR, 3D pre-operative planning, PSI, and electromagnetic tracking systems available for elbow arthroplasty. Despite limited available evidence for applications to the elbow, preliminary studies, which are mostly cadaveric or experimental, show improved implant positioning and satisfactory clinical outcomes. Although it remains uncertain to what extent implant positioning is associated with clinical outcomes and implant survival, advancements toward improved positioning are encouraging. These developments emphasize the potential benefit of these novel technologies in future elbow arthroplasty and may help reduce the disparity in implant survival compared to other types of arthroplasties.

## Disclaimers:

Funding: No funding was disclosed by the authors.

Conflicts of interest: The authors, their immediate families, and any research foundations with which they are affiliated have not received any financial payments or other benefits from any commercial entity related to the subject of this article.
