# Supplementary material for: Technological advancements for surgical planning and intraoperative navigation in elbow arthroplasty: a systematic review
Source: JSES Int. 2026 Aug 5;10(6):101776. doi: 10.1016/j.jseint.2026.101776 (PMC13571538; doi:10.1016/j.jseint.2026.101776)
Supplement: Supplementary file 1 [file mmc1.docx]

**Supplemental File**

| **Supplementary Table 1 - Detailed search string for the used databases** | | | |
| --- | --- | --- | --- |
| **Database  (Platform)** | **Years of coverage** | **Records  (after duplication)** | **Search String** |
| Medline All  (Ovid) | 1946 - Present | 342 (342) | (exp "Augmented Reality"/ OR exp "Virtual Reality"/ OR exp "Robotic Surgical Procedures"/ OR exp "Imaging, Three-Dimensional"/ OR exp "Printing, Three-Dimensional"/ OR exp "Surgery, Computer-Assisted"/ OR "Surgical Navigation Systems"/ OR (VR OR AR OR ((virtual* OR augment* OR mixed OR extended) ADJ6 (realit*)) OR smart-glass* OR smartglass* OR ((AR OR augment*-realit*) ADJ3 (glass* OR head*)) OR google-glass* OR robot* OR 3d* OR 3-d* OR three-dimens* OR threedimen* OR ((computer* OR navigat*) ADJ3 (surg* OR arthroplast* OR hemiarthroplast*)) OR ((stereotactic* OR stereo-tactic*) ADJ3 (neuro-navigat* OR neuronavigat*)) OR ((patient*) ADJ3 (specific*) ADJ3 (instrument*)) OR PSI OR virtual*-environ*).ab,ti,kf.) AND (exp "Elbow"/ OR exp "Elbow Joint"/ OR (elbow* OR articulatio-cubitii* OR cubital-joint*).ab,ti,kf.) AND ("Arthroplasty"/ OR exp "Arthroplasty, Replacement, Elbow"/ OR exp "Elbow Prosthesis"/ OR "Prostheses and Implants"/ OR (arthroplas* OR hemiarthroplast* OR prosthes* OR prosthet* OR ((surgical*) ADJ3 (repair* OR replac*))).ab,ti,kf.) |
| Embase (Embase.com) | 1971 - Present | 573 (294) | ('extended reality'/exp OR 'augmented reality system'/exp OR 'virtual reality system'/exp OR 'robot-assisted procedure'/exp OR 'three-dimensional imaging'/exp OR 'three dimensional printing'/exp OR 'computer assisted surgery'/exp OR 'surgical navigation system'/de OR 'stereotactic neuronavigation system'/exp OR 'patient specific instrumentation'/exp OR 'patient specific instrument'/exp OR (VR OR AR OR ((virtual* OR augment* OR mixed OR extended) NEAR/6 (realit*)) OR smart-glass* OR smartglass* OR ((AR OR augment*-realit*) NEAR/3 (glass* OR head*)) OR google-glass* OR robot* OR 3d* OR 3-d* OR three-dimens* OR threedimen* OR ((computer* OR navigat*) NEAR/3 (surg* OR arthroplast* OR hemiarthroplast*)) OR ((stereotactic* OR stereo-tactic*) NEAR/3 (neuro-navigat* OR neuronavigat*)) OR ((patient*) NEAR/3 (specific*) NEAR/3 (instrument*)) OR PSI OR virtual*-environ*):ab,ti,kw) AND ('elbow'/exp OR 'elbow joint'/exp OR (elbow* OR articulatio-cubitii* OR cubital-joint*):ab,ti,kw) AND ('arthroplasty'/de OR 'elbow arthroplasty'/exp OR 'revision arthroplasty'/exp OR 'prosthesis'/de OR 'elbow prosthesis'/exp OR (arthroplas* OR hemiarthroplast* OR prosthes* OR prosthet* OR ((surgical*) NEAR/3 (repair* OR replac*))):ab,ti,kw) |
| Web of Science Core Collection ^a^ (Web of Knowledge) | 1975 - Present | 602 (349) | (TS=(VR OR AR OR ((virtual* OR augment* OR mixed OR extended) NEAR/5 (realit*)) OR smart-glass* OR smartglass* OR ((AR OR augment*-realit*) NEAR/2 (glass* OR head*)) OR google-glass* OR robot* OR 3d OR 3-d OR 3dimens* OR 3-dimens* OR three-dimens* OR threedimen* OR ((computer* OR navigat*) NEAR/2 (surg* OR arthroplast* OR hemiarthroplast*)) OR ((stereotactic* OR stereo-tactic*) NEAR/2 (neuro-navigat* OR neuronavigat*)) OR ((patient*) NEAR/2 (specific*) NEAR/2 (instrument*)) OR PSI OR virtual*-environ*)) AND (TS=(elbow* OR articulatio-cubitii* OR cubital-joint*)) AND (TS=(arthroplas* OR hemiarthroplast* OR prosthes* OR prosthet* OR ((surgical*) NEAR/2 (repair* OR replac*)))) |
| Chochrane Central Register of Controlled Trials (Wiley) | 1992 - Present | 18 (9) | ((VR OR AR OR ((virtual* OR augment* OR mixed OR extended) NEAR/6 (realit*)) OR smart NEXT/1 glass* OR smartglass* OR ((AR OR augment* NEXT/1 realit*) NEAR/3 (glass* OR head*)) OR google NEXT/1 glass* OR robot* OR 3d* OR 3 NEXT/1 d* OR three NEXT/1 dimens* OR threedimen* OR ((computer* OR navigat*) NEAR/3 (surg* OR arthroplast* OR hemiarthroplast*)) OR ((stereotactic* OR stereo NEXT/1 tactic*) NEAR/3 (neuro NEXT/1 navigat* OR neuronavigat*)) OR ((patient*) NEAR/3 (specific*) NEAR/3 (instrument*)) OR PSI OR virtual* NEXT/1 environ*):ab,ti,kw) AND ((elbow* OR articulatio NEXT/1 cubitii* OR cubital NEXT/1 joint*):ab,ti,kw) AND ((arthroplas* OR hemiarthroplast* OR prosthes* OR prosthet* OR ((surgical*) NEAR/3 (repair* OR replac*))):ab,ti,kw) |
| Google Scholar ^b^ |  | 100 (91) | VR\|'virtual\|augmented\|extended reality' elbow\|elbows arthroplasty\|artheroplasties\|arthroplastic |
| ^a^ Science Citation Index Expanded (1975-present); Social Sciences Citation Index (1975-present); Arts & Humanities Citation Index (1975-present); Conference Proceedings Citation Index- Science (1990-present); Conference Proceedings Citation Index- Social Science & Humanities (1990-present); Emerging Sources Citation Index (2005-present) ^b^ Google Scholar was searched via "Publish or Perish" to download the results in EndNote. | | | |
